# A conserved histone deacetylase with a role in the regulation of cytokinesis in *Schizosaccharomyces pombe*

**DOI:** 10.1186/1747-1028-7-13

**Published:** 2012-05-04

**Authors:** Charnpal Grewal, Jack Hickmott, Stefan Rentas, Jim Karagiannis

**Affiliations:** 1 Department of Biology, University of Western Ontario, London, Ontario N6A-5B7, Canada

**Keywords:** Fission yeast, Cytokinesis, Cell division, Histone deacetylase

## Abstract

**Background:**

In *Schizosaccharomyces pombe* the SET domain protein, Set3p - together with its interacting partners, Snt1p, and Hif2p - form a complex that aids in preventing cell division failure upon mild cytokinetic stress. Intriguingly, the human orthologs of these proteins (MLL5, NCOR2, and TBL1X*)* are also important for the faithful completion of cytokinesis in tissue culture cells. Since MLL5, NCOR2, and TBL1X form a complex with the histone deacetylase, HDAC3, we sought to determine if an orthologous counterpart played a regulatory role in fission yeast cytokinesis.

**Results:**

In this report we identify the *hos2* gene as the fission yeast *HDAC3* ortholog. We show that Hos2p physically interacts with Set3p, Snt1p, and Hif2p, and that *hos2∆* mutants are indeed compromised in their ability to reliably complete cell division in the presence of mild cytokinetic stresses. Furthermore, we demonstrate that over-expression of *hos2* causes severe morphological and cytokinetic defects. Lastly, through recombinase mediated cassette exchange, we show that expression of human *HDAC3* complements the cytokinetic defects exhibited by *hos2∆* cells.

**Conclusions:**

These data support a model in which Hos2p functions as an essential component of the Set3p-Snt1p-Hif2p complex with respect to the regulation of cytokinesis. The ability of human *HDAC3* to complement the cytokinesis defects associated with the deletion of the *hos2* gene suggests that further analysis of this system could provide insight into the role of HDAC3 in both the regulation of cell division, as well as other biological processes influenced by HDAC3 deacetylation.

## Background

In the fission yeast, *Schizosaccharomyces pombe*, regulatory mechanisms exist to ensure that cytokinesis takes place at the correct spatial location within the cell and at the proper temporal position of the cell cycle [1-5]. The proper spatial positioning of the cytokinetic actomyosin ring is controlled by the anilin-related protein, Mid1p, which - upon entry into mitosis - re-localizes from the nucleus to a medial band defining the future site of cell division [5-9]. The initiation of ring constriction, on the other hand, is signalled by a conserved regulatory module referred to as the Septation Initiation Network (SIN). The SIN is composed of a GTPase signalling cascade that is essential for the temporal co-ordination of cytokinesis, ring constriction, and for the deposition of the division septum [3,5,9,10].

 In addition to these mechanisms, recent work has also supported the existence of a cytokinesis monitoring system. This system has the capacity to generate a prolonged cytokinesis competent state (characterized by delayed entry into mitosis and the continuous repair/re-establishment of the actomyosin ring) that allows for the successful completion of cell division upon mild cytokinetic stresses [11-17]. The key components of this regulatory module are the Cdc14 family phosphatase, Clp1p, and the 14-3-3 protein, Rad24p. When challenged by stresses that perturb the cell division machinery, a phosphorylated form of Clp1p (normally nucleolar) becomes bound by Rad24p leading to its prolonged retention in the cytoplasm and the extended activation of the SIN. In the absence of either Clp1p or Rad24p, cells are unable to maintain SIN signalling leading to cytokinesis failure and the generation of inviable, multinucleate cells [13,15,18].

A useful strategy in both identifying these regulators, and in defining their roles, has involved the treatment of fission yeast cells with the actin depolymerising drug, Latrunculin A (LatA) [11-15]. At the concentrations used (20–50 times less than that needed to completely depolymerize the actin cytoskeleton) such treatment impedes constriction of the actomyosin ring and is lethal to both *clp1∆* and *rad24∆* mutants (due to their inability to prolong the cytokinesis competent state). Wild-type cells in contrast, are able to complete cell division under these conditions, albeit at rates slower than in untreated cells.

Interestingly, a recent genome-wide genetic screen based on the isolation of deletion mutants hyper-sensitive to LatA, identified *set3hif2*, and *snt1* and showed that their respective gene-products form a nuclear-localized complex required for the dependable execution of cytokinesis. Further analysis demonstrated that *set3∆* mutants were unable to properly modulate the expression of stress response genes, suggesting a role for the Set3p complex in effecting changes in gene expression required to counter the effects of LatA induced stress [19].

Intriguingly, the *set3snt1*, and *hif2* genes are orthologous to human *MLL5NCOR2* and *TBL1X*, which together encode components of a histone deacetylase complex. Remarkably, knockdown of either of these genes in human HeLa cells results in increased rates of cytokinesis failure [20]. Since NCOR2, and TBL1X physically associate with the type I histone de-acetylase, HDAC3 - a highly conserved histone deacetylase with orthologs from *Dictyostelium* to multicellular mammals - we sought to determine if an orthologous counterpart played a regulatory role in fission yeast cytokinesis [20-22].

Here we identify the *hos2* gene as the fission yeast *HDAC3* ortholog. Hos2p, also known as Hda1p, is a non-essential histone de-acetylase known to affect H4K16 acetylation (primarily in the 5′ end of genes) as well as gene silencing and sporulation efficiency [23-25]. In this report we show that Hos2p exists in a complex with Set3p, Snt1p, and Hif2p, and that *hos2∆* mutants are also compromised in their ability to complete cytokinesis in the presence of low doses of LatA. Furthermore, a role in the regulation of cell division is supported by the severe morphological and cytokinetic defects observed upon *hos2* over-expression.

Lastly, we provide strong support for the conservation of HDAC3 function by demonstrating the ability of human *HDAC3* to complement the cytokinetic defects exhibited by *hos2∆* cells.

## Results

### Hos2p is required for the successful completion of cytokinesis in response to perturbation of the cell division machinery

[20-22] To determine if an ortholog of HDAC3 existed in *S. pombe*, and if it too played a role in the regulation of cytokinesis, A BLAST search using human HDAC3 as query was performed. This analysis revealed strong conservation of amino acid sequence between HDAC3 and fission yeast Hos2p (not be confused with the DASH complex subunit, Dad2p, which is also sometimes referred to using the gene name, *hos2*). The proteins share 51% identity (63% similarity), are of similar length (427 and 437 aa, respectively), and possess a single histone deacetylase domain (PFAM00850) that comprises almost the entire length of the protein (Additional File 1).

To determine if Hos2p played a role in cytokinesis, the *hos2* gene deletion mutant was purchased from the commercial supplier, Bioneer. After confirmation of the deletion via colony PCR, wild-type and *hos2∆* strains were grown to mid-log phase and serial dilutions plated onto YES media containing either 0.5 μM LatA or DMSO (solvent control).

**Figure 1:**
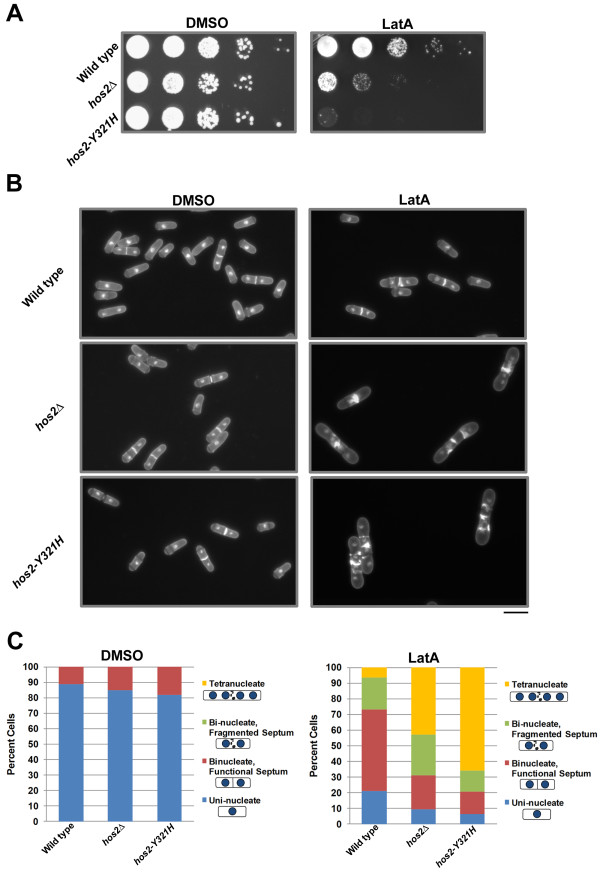
***hos2 ****∆ ***and*hos2-Y321H *mutants are hyper-sensitive to LatA treatment. (A)** Ten-fold serial dilutions of logarithmically growing cells of the indicated genotype were plated onto YES plates containing 0.5 μM LatA or DMSO (solvent control) at 30°C for 3 d. **(B)** Cells of the indicated genotype were grown to mid-log phase at 30°C and then treated with 0.5 μM LatA for 5 h before being fixed and stained with DAPI (nuclei) and aniline blue (cell wall/septa). Bar, 10 μm. (**C)** Quantitation of phenotypes of cells treated as in B. Between 200 and 500 cells were counted for each genotypic class.

Interestingly, the *hos2∆* strain demonstrated a substantial decrease in viability when grown in the presence of LatA. In contrast, while the rate of growth of wild-type cells decreased in LatA media, viability was not affected (note the formation of small colonies even at the lowest dilution) (Figure 1A).

To determine if the sensitivity to LatA was related to defects in cytokinesis, both wild-type and *hos2∆* strains were grown in liquid YES media and then treated with either 0.5 μM LatA or DMSO for 5 hours at 30°C. Cells were then fixed and stained with DAPI and analine blue to visualize nuclei and cell wall/septal material, respectively. No obvious morphological or cytokinesis phenotypes were observed in *hos2∆* cells under normal growth conditions. However, in LatA media, *hos2∆* mutants were severely impaired in their ability to complete cell division and accumulated a large proportion of tetra-nucleate cells with fragmented septa. In contrast, the majority of wild-type cells were bi-nucleate and formed functional, albeit thickened and sometimes malformed septa (Figure 1B).

To quantitate the data, cells were classified into four different phenotypic categories:i) uni-nucleate cells, ii) bi-nucleate cells with a functional septum (i.e. the septum completely bisects the cell), iii) bi-nucleate cells with a fragmented septum (i.e. the septum is non- functional and does not completely bisect the cell), and iv) tetra-nucleate cells. This analysis revealed that while over 40% of *hos2∆* cells were tetra-nucleate, only 6% of wild-type cells showed a similar phenotype. Moreover, while 72% of wild-type cells were either mono- nucleate, or bi-nucleate (with a functional septum), only 31% of *hos2∆* cells were similarly distributed (Figure 1C). The phenotypes observed in *hos2∆* mutants upon LatA treatment are unlikely to be due to defects in SIN activity since two independent markers of active SIN signalling - Cdc7p localization to a single SPB, and increased export of Clp1p from the nucleolus to the cytoplasm [15] - were normal in *hos2∆* mutants upon exposure to LatA (Additional File 2).

An important role for Hos2p in responding to LatA treatment was also supported by synthetic genetic interactions between the *hos2∆* and *clp1∆* mutations. Since Clp1p is required for the function of the cytokinesis checkpoint, weak cytokinesis mutants often display stronger phenotypes in *clp1∆* backgrounds [15]. To test if this were true in the case of *hos2∆* mutants, *clp1∆hos2∆* and *clp1∆ hos2∆* mutants were examined after treatment with both 0.1 μM and 0.5 μM LatA. Interestingly, while *hos2∆* and *clp1∆* single mutants were viable at 0.1 μM LatA, double mutants displayed severe cytokinesis defects at this concentration (Additional File 3).

Lastly, we also noted that the presence of the *hos2∆* mutation was capable of lowering the restrictive temperature of the *ts cdc15-140* mutation by ~ 2°C (*cdc15* encodes an F-BAR protein required for contractile ring assembly; [26]) (Additional File 4). Interestingly, this decrease in the restrictive temperature of the *cdc15-140* mutation is similar to that caused by the presence of the *set3∆snt1∆*, and *hif2∆* gene deletions in *cdc15-140* backgrounds. These data further support a common function for the *hos2set3snt1*, and *hif2* genes [19].

Once having established that the Hos2p protein was indeed involved in the regulation of cytokinesis, we explored the possibility that the protein’s deacetylase activity was related to its function. To this end we created a mutant strain expressing a form of Hos2p in which the catalytically active tyrosine residue in the catalytic pocket was replaced with a catalytically inactive histidine residue (Y321H). Interestingly, the cytokinesis phenotypes of this mutant were more severe than those displayed by *hos2∆* cells. Cells bearing the Y321H mutation were almost completely inviable in the presence of LatA and furthermore, over 60% were tetra-nucleate after 5 hours growth in liquid media containing 0.5 μM LatA (Figure 1A-C).

**Figure 2:**
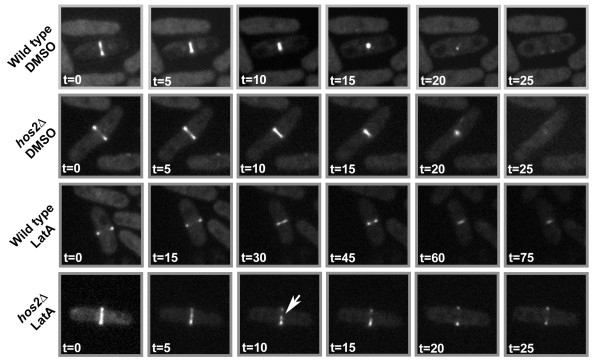
***hos2 ****∆***cells are unable to maintain the integrity of the actomyosin ring upon LatA treatment.** Wild-type and *hos2∆* cells expressing the Rlc1-GFP fusion protein (marker of the actomyosin ring) were imaged upon treatment with 0.5 μM LatA or DMSO using the CellAsics ONIX Microfluidic Perfusion Platform. Images were taken every 30 s. Arrow indicates fragmented ring.

To more closely examine the effects of LatA on cytokinesis we created a *hos2∆* strain expressing a marker of the actomyosin ring, Rlc1-GFP [27]. Using the CellAsics ONIX Microfluidic Perfusion Platform, we were able to monitor the constriction of the actomyosin ring while perfusing liquid YES media containing either 0.5 μM LatA or DMSO as a solvent control. In DMSO media, wild-type cells were able to fully constrict the ring in approximately ~25 minutes. Similarly, rings in *hos2∆* mutants grown in DMSO media displayed comparable kinetics and were also able to fully constrict in ~25 minutes (Figure 2, top two rows; Additional Files 5 and 6). However, when grown in LatA media, *hos2∆* cells displayed dramatic differences in phenotype compared to wild type. The majority of wild-type cells were able to form and constrict the ring in the presence of LatA, albeit over a much longer time frame (~90 minutes) than DMSO controls (7 out of 9 cells). The remainder (2 out of 9 cells) were able to maintain the integrity of the ring over this time frame, but were not able to fully constrict the ring over the 90 minute time-lapse. In contrast, *hos2∆* cells could not preserve the physical integrity of the ring. In LatA treated *hos2∆* mutants, Rlc1- GFP signal did not constrict and instead appeared to fragment (8 out of 8 cells) within 10–15 minutes (Figure 2, bottom two rows; Additional Files 7 and 8).

### Over-expression of Hos2p results in severe morphological and/or cytokinetic defects

To explore whether Hos2p played a dosage dependent role in cytokinesis we decided to test the effects of *hos2* over-expression using the pREP series of thiamine repressible expression vectors [28,29]. To this end, full length *hos2* was cloned downstream of the *nmt1/41/81* promoters present within the pREP1/41/81 plasmids and the constructs were transformed into wild-type strain, JK484 (Table 1). Full strength expression is obtained from the *nmt1* promoter, while *nmt41* and *nmt81* promoters contain site mutations that decrease expression to intermediate and low levels, respectively [28].

**Table 1:** Strains used in this study

**Strain Name**	**Relevant Genotype**	**Source**
JK9	*clp1::ura4*^*+*^*ura4-D18 h*^*-*^	JK Collection
JK468	*hos2::KanMX4 ura4-D18 leu-32 ade6-216*	Bioneer
JK484	*ura4-D18 leu*‐*32 ade6*‐*216 his3-D1*	JK Collection
JK561	*hos2-GFP::ura4*^*+*^*ura4-D18 leu1-32 ade6- 210 his3-D1*	This Study
JK648	*hos2-Y321H::ura4*^*+*^*ura4-D18*	This Study
JK659	*rlc1GFP::ura4*^*+*^*hos2::kanMX4 ura4-D18*	This Study
JK737	*hos2::ura4+ ura4-D18 leu*‐*32 ade6*‐*216 his3-D1* (base strain)	This Study
JK744	*hos2::hos2*^*Sp*^*ura4-D18 leu*‐*32 ade6*‐*216 his3-D1*	This Study
JK745	*hos2::HDAC3*^*Hs*^*ura4-D18 leu*‐*32 ade6*‐*21 6his3-D1*	This Study
JK759	*hos2::kanMX4 clp1::ura4*^*+*^*ura4-D18*	This Study
JK761	*hos2::kanMX4 cdc15-140 ade6-21x leu1-32*	JK Collection
JK776	*cdc7GFP::ura4*^*+*^*hos2::ura4*^*+*^*ura4-D18*	This Study
JK778	*clp1GFP::kanMX4 hos2::ura4+ ura4-D18*	This Study
MBY154	*cdc15-140 ade6-21x leu1-32 h*^*-*^	JK Collection
MBY624	*rlc1GFP::ura4*^*+*^*ura4-D18 h*^*+*^	JK Collection
MBY978	*clp1GFP::kanMX4 ura4-D18 leu1-32 ade6-216 h+*	JK Collection
MBY2415	*cdc7GFP::ura4*^*+*^*ura4-D18 ade6-21x leu1-32 ura4-D18 his3-D1 h+*	JK Collection
SCG5	*hos2-myc::ura4*^*+*^*ura4-D18 leu1-32 ade6-210 his-D1*	This Study
SCG10	*set3-HA::ura4*^*+*^*hos2-myc::ura4*^*+*^*ura4-D18*	This Study
SCG11	*snt1-HA::ura4*^*+*^*hos2-myc::ura4*^*+*^*ura4-D18*	This Study
SCG14	*hif2-HA::ura4*^*+*^*hos2-myc::ura4*^*+*^*ura4-D18*	This Study

**Figure 3:**
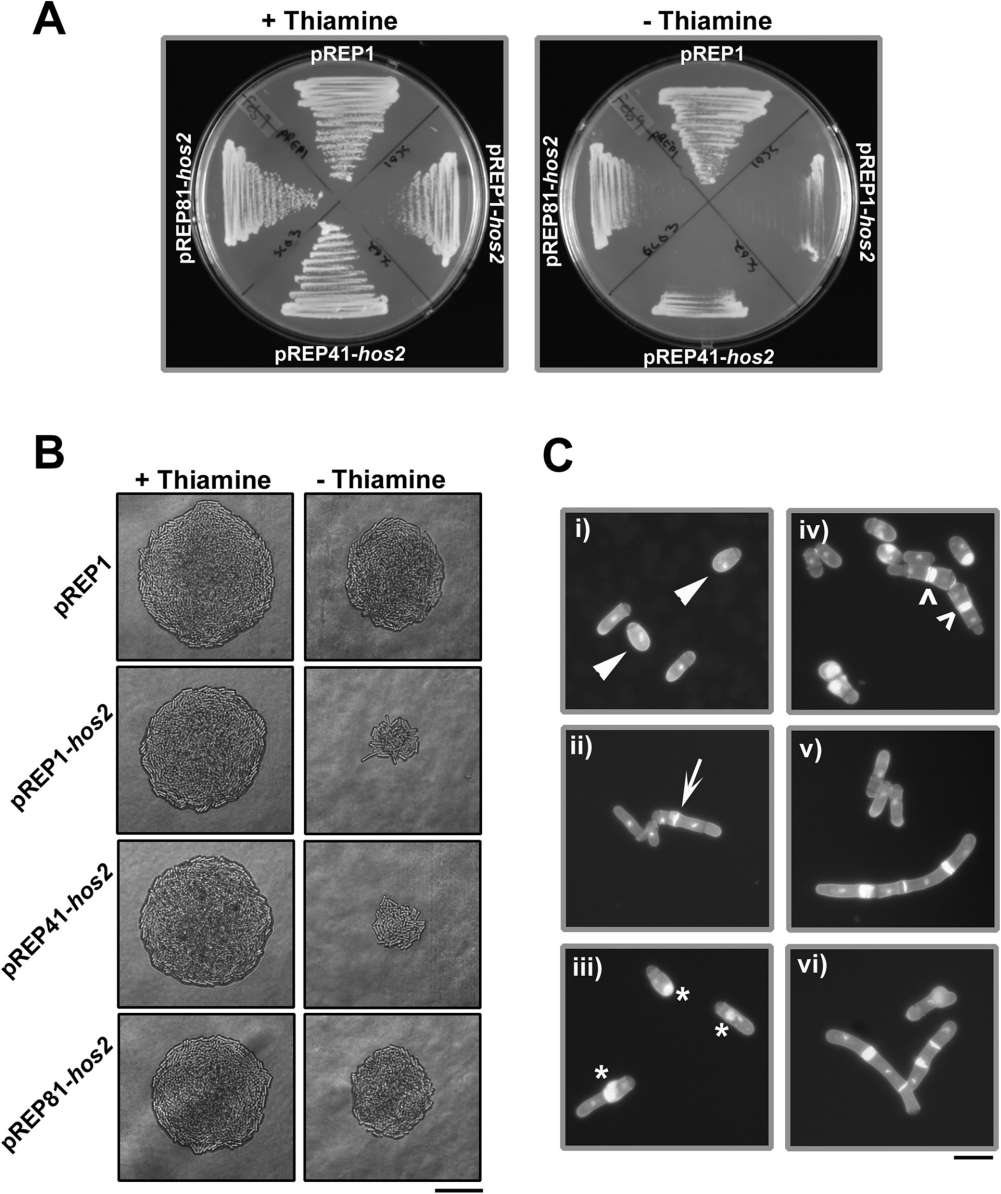
**Over-expression of Hos2p results in growth inhibition and in phenotypic defects related to morphogenesis and/or cytokinesis. (A)** Strains expressing *hos2* from the *nmt1/41/81* series of promoters were streaked to EMM media in the presence (repressed) or absence (de-repressed) of thiamine and incubated at 30°C for two days. **(B)** Colony morphology of strains treated as in A. Bar, 50 μm **(C)** Representative examples of the pleiotropic cellular morphology exhibited by cells expressing *hos2* from the *nmt1* promoter; (i) cells with a rounded, de-polarized appearance, ii) cells with misplaced septa, iii) cells with abnormally excessive and mis-localized septal deposition, iv) cells with multiple septa v) highly elongated cells containing multiple aberrant deposits of septal material, vi) elongated and branched cells. Bar, 10 μm.

In the presence of thiamine (repressed) cells expressing *hos2* from either *nmt41*, or *nmt81* promoters (as well as an empty vector control) displayed normal growth whereas cells expressing *hos2* from the *nmt1* promoter showed slight growth inhibition (most likely due to the fact that expression from *nmt1* is somewhat “leaky”) [28,29]. On the other hand, when grown in the absence of thiamine (de-repressed) cells expressing *hos2* displayed an inhibition of growth ranging from severe (in the case of the *nmt1* promoter) to intermediate (in the case of the *nmt41* promoter) to mild (in the case of the *nmt81* promoter) (Figure 3A, B).

Interestingly, expression of *hos2* from the *nmt1* promoter also led to a series of unusual and pleiotropic phenotypes related to morphogenesis and/or cytokinesis. While the majority of cells appeared normal, others displayed phenotypes ranging from i) slight morphological abnormalities such as a rounded, de-polarized appearance, ii) misplaced, but otherwise normal septa, iii) abnormally excessive and mis-localized septal deposition, iv) multiple septa v) highly elongated cells containing multiple aberrant deposits of septal material and vi) highly unusual branched and elongated cells (Figure 3C; Table 2). This broad range of phenotypes further supports a model where Hos2p plays a role in the regulation of cytokinesis and/or morphogenesis.

**Table 2:** **Quantitation of phenotypic abnormalities upon *hos2 *or *HDAC3 *over-expression from the *nmt1 *promoter in the absence of thiamine**

**Morphological Abnormality**	**pREP1**	**pREP1-*hos2***	**pREP1-*HDAC3***
Normal	> 99%	52%	54%
Rounded/Depolarized	< 1%	26.5%	29%
Misplaced septa	<1%	9%	5%
Excessive/Misplaced septal deposits	0	6%	6%
Multiple septa	0	2%	2%
Elongated with multiple aberrant deposits of septal material	0	3%	2%
Elongated and Branched	0	1.5%	1.5%

### Hos2p is nucleo-cytoplasmic and physically interacts with Set3p, Snt1p, and Hif2p

**Figure 4:**
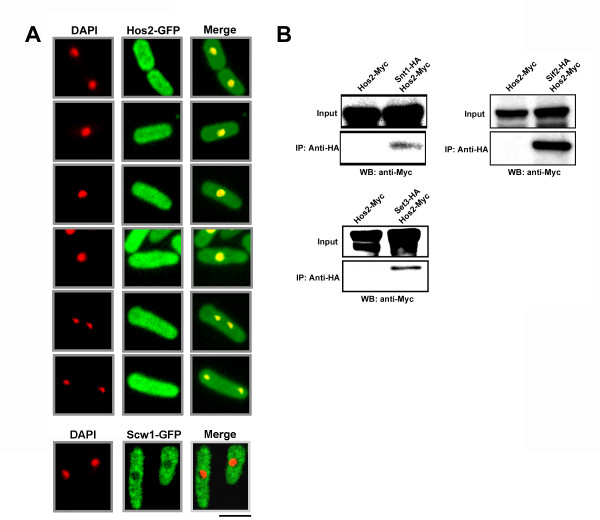
**Hos2p localizes to both the cytoplasm and the nucleus and physically interacts with Set3p, Snt1p, and Hif2p.(A)** Cells expressing the Hos2-GFP fusion protein were grown to mid-log phase at 30°C in YES media, fixed, and then stained with DAPI and observed using the DAPI and GFP filter sets. Bar, 5 μm. **(B)** Cells expressing the indicated fusion proteins were grown to mid-log phase in YES, lysed under native conditions, and subjected to anti-HA immunoprecipitations. Both total lysates and immunoprecipitates were resolved by SDS-PAGE and immunoblotted with antibodies specific for the Myc epitope.

We have previously shown that the Set3p, Snt1p, and Hif2p form a nuclear-localized complex. If indeed a member of this complex, Hos2p would be expected to localize (at least in part) to the nucleus. To test this prediction we created a strain expressing a C- terminal Hos2-GFP fusion protein under control of the native *hos2* promoter. Hos2p localized to both the cytoplasm and to the nucleus as judged by co-staining with the nuclear dye, DAPI (Figure 4A). Furthermore, we determined that the intracellular distribution of Hos2p was not affected by LatA treatment (data not shown), nor did its localization change as a function of cell cycle position (Figure 4A). The localization of the Scw1p protein (enriched in the cytoplasm relative to the nucleus) was used as a control to ensure the validity of the observed nuclear signal (Figure 4A, bottom panels) [30].

To determine if Hos2p was able to physically interact with members of the Set3p complex, in-vivo co-immunoprecipitation experiments using Myc or HA epitope tagged alleles were performed. Significantly, Hos2-Myc fusion proteins could be detected in anti- HA immuno-precipitates of Snt1-HA Hos2-Myc, Hif2-HA Hos2-Myc, and Set3-HA Hos2- Myc extracts, but not in extracts expressing only Hos2-Myc (Figure 4B). These results are consistent with high throughput proteomics experiments aimed at defining fission yeast protein complexes related to histone modification [31]. Taken together, these data suggest that Hos2p molecules can indeed exists within a complex with Snt1p, Hif2p, and/or Set3p.

### Expression of human HDAC3 complements the cytokinesis defects associated with the *hos2* gene deletion

**Figure 5:**
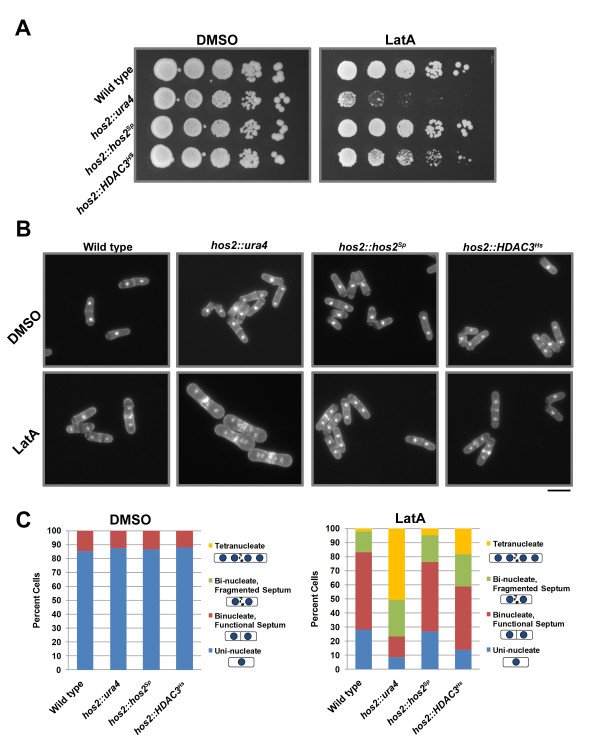
**Expression of human *HDAC3 *complements the cytokinesis defects exhibited by *hos2 ****∆*** mutants. (A)** Ten-fold serial dilutions of logarithmically growing cells of the indicated genotype were plated onto YES plates containing 0.5 μM LatA or DMSO (solvent control) at 30°C for 3 d. **(B)** Cells of the indicated genotype were grown to mid-log phase at 30°C and then treated with 0.5 μM LatA for 5 h before being fixed and stained with DAPI (nuclei) and aniline blue (cell wall/septa). Bar, 10 μm. **(C)** Quantitation of phenotypes of cells treated as in B. Between 200 and 500 cells were counted for each genotypic class.

To further explore the possibility of functional conservation between human HDAC3 and fission yeast Hos2p, we utilized the technique of recombinase mediated cassette exchange to replace the endogenous Hos2p open reading frame in fission yeast with the human *HDAC3* gene (Additional File 9) [32]. This approach first required the creation of a “base strain” in which the *hos2* gene was replaced with a deletion cassette composed of the *ura4*^+^ selectable marker flanked by the Cre recombinase recognitions sites, loxP and loxM3. As expected, the *hos2∆* “base strain” created in this manner was indistinguishable from the Bioneer gene deletion mutant in terms of its sensitivity to LatA and its ability to complete cytokinesis in liquid LatA media (Figure 5A-C).

Next, we cloned the *HDAC3* cDNA from the pOTB7 plasmid (purchased from Open Biosystems) into the *XhoI* and *SacI* sites of the “exchange” plasmid pAW8X. The exchange plasmid contains the Cre recombinase gene under control of the *nmt41* promoter. Once transformed into the base strain, expression of the recombinase permits exchange of the *ura4*^+^ cassette with the *HDAC3* sequence (Additional File 9). In this way the *hos2::HDAC3*^*Hs*^ (Hs, *Homo sapiens*) strain - in which the human HDAC3 gene is under control of the *S. pombe hos2* promoter - was created. In addition a control strain, in which the *S. pombe hos2* gene was re-engineered back into the base strain, *hos2::hos2*^*Sp*^ (Sp, *Schizosaccharomyces pombe*) was used as a control.

Remarkably, expression of human *HDAC3* in *S. pombe* was able to both restore viability and substantially complement the cytokinesis phenotypes characteristic of the base strain, albeit not to the same extent as the re-introduced *hos2* gene (Figure 5A-C). While the base strain displayed over 50% tetra-nucleate cells after 5 hours, the *hos2::hos2*^*Sp*^ strain displayed only 5% tetra-nucleate cells and the *hos2::HDAC3*^*Hs*^ strain only 18% tetra-nucleate cells. Thus, expression of the human *HDAC3* is indeed capable of at least partially complementing the loss of the *S. pombe hos2* gene.

**Figure 6:**
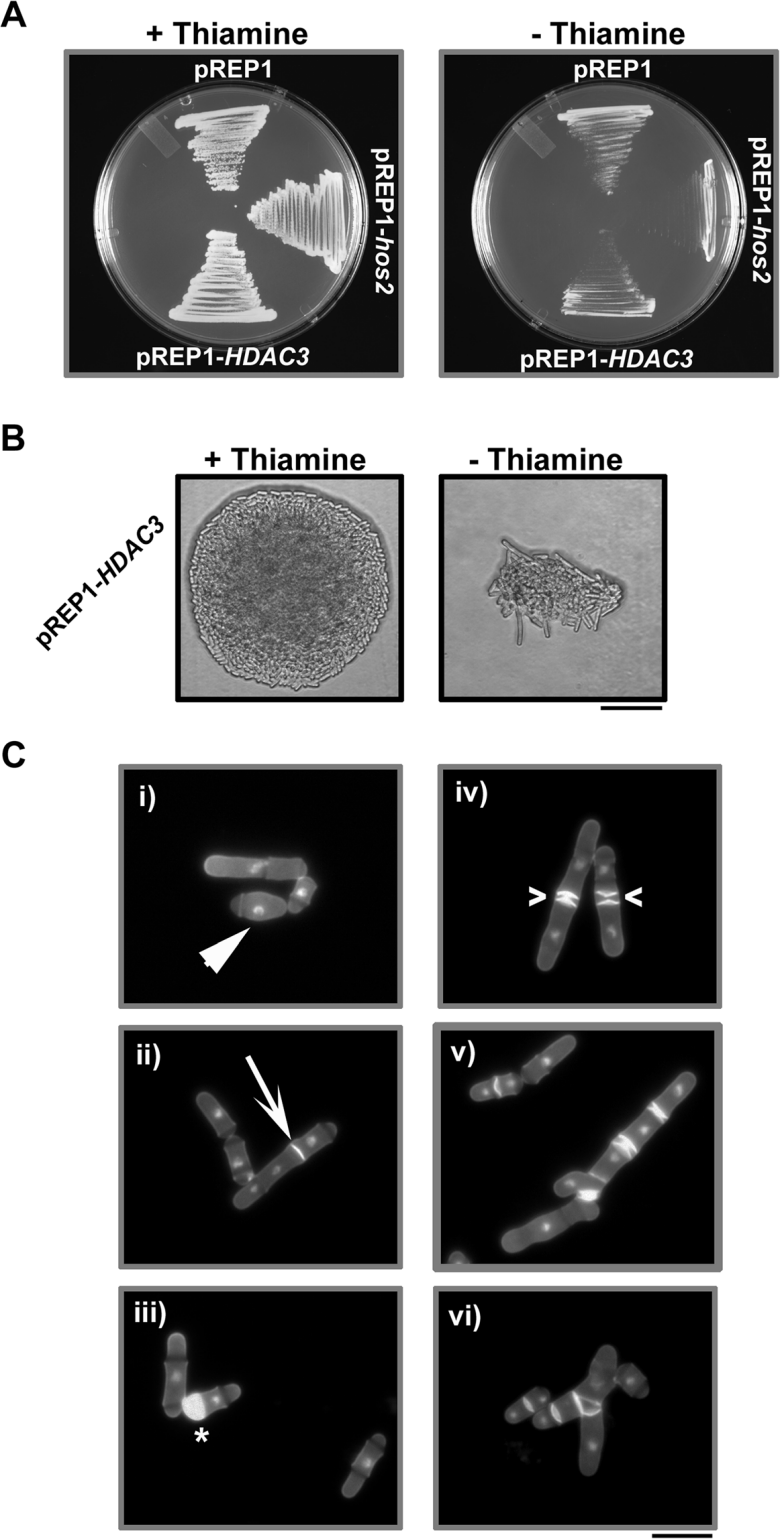
**Similarly to the over-expression of Hos2p, over-expression of human *HDAC3 *results in growth inhibition and in phenotypic defects related to morphogenesis and/or cytokinesis. (A)** Strains expressing *hos2* or *HDAC3* from the *nmt1* promoter were streaked to EMM media in the presence (repressed) or absence (de-repressed) of thiamine and incubated at 30°C for two days. **(B)** Colony morphology of strains treated as in A. Bar, 50 μm **(C)**. Representative examples of the pleiotropic cellular morphology exhibited by cells expressing *HDAC3* from the *nmt1* promoter; (i) cells with a rounded, de-polarized appearance, ii) cells with misplaced septa, iii) cells with abnormally excessive and mis-localized septal deposition, iv) cells with multiple septa v) highly elongated cells containing multiple aberrant deposits of septal material, vi) elongated and branched cells. Bar, 10 μm.

Lastly, to provide further evidence of conserved function, we proceeded to over- express human HDAC3 under the control of the *nmt1* promoter using the pREP1 plasmid. Similar to the over-expression of *hos2*, over-expression of HDAC3 resulted in a decrease in growth rate in the absence of thiamine (de-repressed) and similar morphological and cytokinesis phenotypes (compare Figure 3B,C with Figure 6B,C). Just as with the case of the *hos2* gene, over-expression of *HDAC3* resulted in a similar range of pleiotropic phenotypes including i) cells with a rounded, de-polarized appearance, ii) misplaced septa, iii) abnormally excessive and mis-localized septal deposition, iv) multiple septa v) highly elongated cells containing multiple aberrant deposits of septal material and vi) elongated and branched cells (Figure 6B,C; Table 2). Taking all data together, these results suggest that Hos2p and human HDAC3 function in a similar manner and modulate similar biological processes in *S. pombe*.

## Discussion

While the absence of cytokinesis is tolerated under certain specialized circumstances (e.g. the development of *Drosophila* embryos) it is normally essential for cellular proliferation, differentiation and for maintaining control over ploidy [1,2,33]. Thus, knowledge of the regulatory networks governing cytokinesis is an important component of our basic understanding of eukaryotic cell biology. Moreover, aspects of this knowledge related to cytokinesis failure may also be relevant to our understanding of the mechanisms important for maintaining genomic integrity.

A relationship between cytokinesis failure and genome integrity (and thus tumourigenesis) was first proposed by Theodor Boveri in 1914 [34]. In his classic manuscript “Concerning the origin of malignant tumours” Boveri hypothesized that tetraploid intermediates derived from cytokinetic failure might undergo chaotic multipolar mitoses leading to numerical and/or structural chromosomal defects. Recent experimental evidence (reviewed in [33,35,36]) provides strong support for Boveri’s assertions; most notably the observation that tetraploid mouse mammary epithelial cells (generated by the inhibition of cytokinesis) display increased rates of aneuploidy and give rise to malignant tumours when transplanted into nude mice [37].

Interestingly, a recent genome-wide RNAi screen, aimed at discovering gene-products important for the fidelity of cell division in HeLa cells, identified physical interactors of HDAC3 as being important for cytokinesis [20]. In this work Kittler et al., showed that knockdown of *MLL5NCOR2*, or *TBL1X* (genes encoding putative chromatin-binding proteins implicated in transcriptional regulation) resulted in increased rates of cytokinesis failure and the generation of tetraploid intermediates [20]. Remarkably, these genes are orthologous to three fission yeast genes (*set3snt1*, and *hif2*) recently identified as playing a role in the reliable execution of cytokinesis and which physically interact with the *S. pombe* HDAC3 ortholog, Hosp2p (Figure 4B). These results not only further validate the use of *S. pombe* as a eukaryotic model, but also raise the intriguing question of whether these orthologous complexes represent an evolutionarily conserved module important for the faithful execution of cell division.

In this report we identify the fission yeast ortholog of HDAC3 and provide further evidence of cross-species conservation. First, the expression of human HDAC3 clearly complements the cytokinesis defects displayed by *hos2∆* mutants (Figure 5). Second, over- expression of Hos2p or HDAC3 both result in phenotypes related to morphogenesis and/or cytokinesis (Figure 6). With respect to functional conservation, it is also important to note the similarity in localization between Hos2p and HDAC3. HDAC3 is the only member of the class I HDACs to localize to the cytoplasm, as well as the nucleus, owing to the presence of both nuclear import and export signals [38]. Thus, our observation that Hos2p localizes to both of these intracellular compartments represents another level at which Hos2p and HDAC3 share similarities (Figure 4A). Furthermore, the cytoplasmic localization observed for Hos2p is consistent with immunofluorescence data demonstrating that HA epitope tagged Hos2p is predominantly cytoplasmic as well as with global GFP-fusion based localisation studies showing both nuclear and cytoplasmic localization [39,40].

We also provide genetic evidence supporting a catalytic role for Hos2p through the analysis of strains bearing the Y321H mutation. The mutated tyrosine residue catalyzes stabilization of the transition state between acetyl-lysine and lysine thereby allowing for catalysis of lysine deacetylation; this is not chemically possible through a histidine residue [41]. As expected if Hos2p played a catalytic role, *hos2-Y321H* mutants displayed phenotypes similar to those exhibited by *hos2∆* strains. In fact, the severity of the cytokinesis defects was greater in the site-mutant compared to the gene deletion (Figure 1). We speculate that the increase in severity of the phenotype may be related to the presence of the mutant protein interfering with other components of the pathway in a dominant negative fashion.

While targeted deacetylation is likely an important aspect of Hos2p function, the physiological substrates of the Hos2p-Set3p-Snt1p-Hif2p complex remain unknown. One possibility is that cytokinetic failure is related to transcriptional defects stemming from the abnormal acetylation of histones. A role in transcription is supported by the observation that *set3∆* mutants are compromised in their ability to alter the expression of stress response genes upon LatA treatment. Thus, cell division failure may be a manifestation of the inability of the mutants to properly counter the effects of LatA induced stress leading to the direct and/or indirect effects on the function of the cytokinetic machinery. When considering such models it should also be noted that the substrate specificity of HDACs is not restricted to histones. In fact, many non-histone substrates, including transcription factors, signalling molecules, and nuclear import factors have been identified as targets [42]. Thus, the possibility exists that the complex may function through both the modulation of transcription and/or the post modification of non-histone targets.

Finally, while highly speculative, it is interesting to note that the liver specific deletion of HDAC3 results in hepatocellular carcinoma in mice [43]. In this work, 20 out of 20 *HDAC3*^*−/−*^ displayed low grade hepatocellular carcinoma at a mean age of 10.2 months. Although a role for cytokinesis failure in this phenotype has not been examined, it is interesting to speculate as to whether it played any role in tumour development in this context. Regardless, the demonstration of functional conservation between HDAC3 and Hos2p suggests that further analysis of these proteins in fission yeast might translate into a theoretical framework for understanding the role of HDAC3 in both the regulation of cytokinesis as well as other biological processes influenced by HDAC3 deacetylation.

## Conclusions

In this work we demonstrate a role for the histone deacetylase, Hos2p, in promoting the faithful and dependable execution of cytokinesis in fission yeast. Analysis of catalytically inactive *hos2-Y321H* mutants suggests that its role in this process is mediated through its deacetylase activity. In addition, co-immunoprecipitation experiments indicate that Hos2p regulates cytokinesis as part of a complex with Set3p, Snt1p, and Hif2p (all of which play a documented role in preventing cell division failure). Lastly, the ability of human HDAC3 to complement the cytokinesis defects exhibited by *hos2∆* mutants suggests that continued analysis of this system could translate into a theoretical framework for understanding how HDAC3 functions in more developmentally complex organisms.

## Methods

### Yeast methods

All *Schizosaccharomyces pombe* strains used in this study are listed in Table 1. Strains were derived from the Karagiannis lab collection, constructed during the course of this work, or purchased from Bioneer Corporation (Alameda, CA). *S. pombe* cells were cultured in YES or in Edinburgh Minimal Media (EMM) supplemented with adenine, histidine, leucine, and/or uracil. Liquid cultures were grown with shaking (200 rpm) at 30°C [44]. In experiments involving Latrunculin treatment, *S. pombe* cells were grown to mid log phase (O.D. 0.2) and treated with 0.1-0.5 μM of Latrunculin A (Enzo Life Sciences International, Plymouth Meeting, Pennsylvania) dissolved in DMSO. Cells were grown at 30°C with shaking at 200 rpm for 3–6 hrs, before being fixed with ethanol and stored in PBS pH 7.4. All experiments were repeated a minimum of three times. Plasmid vectors were transformed into *S. pombe* using the lithium acetate protocol according to Forsburg and Rhind [44].

### Fluorescence microscopy

To observe nuclei and cell wall/septa material, cells were prepared as described above (see Yeast Methods) and mixed with 0.02 μg/μL 4′6,-diamidino-2-phenylindole (DAPI) and 1 μg/μL aniline blue. In colocalization experiments, *S. pombe* cells expressing the Hos2-GFP fusion were fixed with ethanol and stored in PBS pH 7.4 To observe nuclei and cell wall/septa material, cells were mixed with DAPI and 1 μg/μL aniline blue prior to observation with the DAPI and GFP filter sets. In time-lapse experiments log-phase cells expressing the Rlc1-GFP fusion were imaged live using the CellAsics ONIX™ Microfluidic Perfusion Platform while perfusing liquid YES growth medium containing 0.5 μM LatA or DMSO as a solvent control. All Images were acquired using a Leica DMI6000B inverted microscope equipped with a 100X Plan Apochromat 1.4 NA Oil objective, a Photometrics QuantEM:512SC EMCCD camera, in conjunction with a BDCARVII spinning disk confocal imager (Z-resolution of 0.5 μm) driven by Metamorph software.

### Cloning methods

*S. pombe* strains expressing carboxy-terminal epitope tagged fusion proteins were constructed using a PCR based cloning strategy. To create the Hos2-GFP and Hos2-Myc expressing strains a C-terminal fragment of the *hos2* gene was PCR amplified using High- Fidelity PCR Enzyme Mix (Fermentas Life Sciences) from *S. pombe* genomic DNA with the forward primer 5’-GGG GGG AAT TCT GAA CGA ATT TTT CGC ACC AGA T-3′ and reverse primer 5′-GGG GGC CCG GGG CCT CGA ACG CGA ACA TC-3′ and cloned in frame into the *EcoRI* and *SmaI* sites of the pJK210-GFP and pJK210-Myc vectors respectively. Molecular cloning of the desired C-terminal fragments was confirmed by restriction digestion and DNA sequencing. Plasmid clones containing the desired C-terminal fragment were transformed into *S. pombe* strain MBY1343 (*ura4-D18*). Ura4^+^ integrants were selected for by growth on EMM lacking uracil and subjected to colony PCR to identify clones in which the construct had integrated into the genome via homologous recombination. HA epitope-tagged versions of Set3p, Hif2p, and Snt1p were created as described earlier.

Thiamine repressible *hos2* expression plasmids were created by PCR amplifying the *hos2* gene (forward primer: 5′-GGG GGA TTA ATA TGG ATA CTC CTG AGA CAT CCA CAC-3′; reverse primer: 5′-GGG GGG GAT CCT CAG CCT CGA ACG CGA AC-3′) from wild-type genomic DNA and cloning into the *NdeI* and *BamHI* sites of pREP1/41/81. Plasmids were then transformed into strain JK484 (Table 1) using the LiAc method and Leu + transformants selected in EMM media supplemented with adenine, uracil, and histidine.

Plasmid bearing strains were grown in supplemented EMM + thiamine overnight, washed three times with EMM media, and then cultured for 24 hrs. Cells were then fixed with ethanol, stained with DAPI and analine blue, and imaged using a Leica DMI6000B microscope driven by Metamorph software using the DAPI filter set.

To determine the effects of HDAC3 over-expression, the full length human *HDAC3* cDNA, was PCR amplified (forward primer: 5′-GAT CGA TTA ATA TGG CCA AGA CCG TGG CC-3′; reverse primer: 5′-CGA TCG GAT CCT TAA ATC TCC ACA TCG CTT T-3′) from the pOTB7 plasmid (Open Biosystems) and cloned into the *NdeI* and *BamHI* sites of the pREP series of plasmids. The plasmids were then transformed into JK484 (Table 1) and the effects of over-expression examined as described above.

To create the *hos2-Y321H* mutant, an N-terminal fragment of the *hos2* gene was PCR amplified with a reverse primer incorporating the Y321H mutation (forward primer #1: 5′- GGG GGG AAT TCT GAC GTG GTG AGG CTA GTG GAT TC-3′; reverse primer #1: 5′- TTT CTA AGA GTA TGA CCA CCA CCT C-3′). Similarly, a C-terminal fragment of the *hos2* gene was PCR amplified with a forward primer incorporating the Y321H mutation (forward primer #2: 5′-GAG GTG GTG GTC ATA CTC TTA GAA A-3′; reverse primer #2: 5′-GGG GGC CCG GGG CCT CGA ACG CGA ACA TC-3′). A full-length product containing the desired mutations was then PCR amplified using forward primer #1, reverse primer #2, and the products of the first two reactions as template. The full-length product was then cloned into the *EcoRI* and *SmaI* sites of the pJK210 vector and the construct transformed into JK484 (Table 1) to obtain integrants. Ura + transformants were then isolated and subjected to colony PCR to identify clones in which the construct had integrated into the genome via homologous integration.

### Biochemical and immunological methods

Cells of the indicated genotype were grown to the mid-log phase at 30°C, collected by centrifugation, and resuspended in STOP buffer (10 mM Tris–HCl pH 8.0, 150 mM NaCl, 50 mM NaF, 10 mM EDTA, 1 mM NaN_3_). Cell pellets were stored at −80°C up to a maximum of 6 months. Cell pellets were subsequently thawed, and lysed using vortexing with glass beads in extraction buffer (1% IGEPAL CA630 (tetr-Octylphenoxy polyethanol), 150 mM NaCl, 50 mM Tris–HCl pH 8.0, 2 mM EDTA, 1 mM PMSF (phenylmethanesulphonylfluoride), 2 mM Benzamidine, 50 mM NaF, 0.1 mM Na_3_VO_4_, 50 mM B-glycerophosphate, 15 mM p-nitrophenyl phosphate, ¼ Tablet Sigma Protease Inhibitors). Immunoprecipitations were performed using Protein G Dynabeads® (Invitrogen) according to the manufacturer’s protocol. Briefly, anti-HA antibodies (HA.11; Sigma) were incubated with Protein G Dynabeads in extraction buffer. Cell extracts were then added to the antibody-bound bead slurry. After incubation and repeated washing with extraction buffer, the bound proteins were eluted by incubation at 96°C for 5 minutes. The eluted proteins were then subjected to SDS-PAGE, transferred to PVDF membranes and probed with anti-Myc antibodies (9E10; Sigma).

### Recombinase mediated cassette exchange

The *hos2* “base” strain was created by deleting the *hos2* coding sequence with the loxP-*ura4*^+^-loxM3 deletion cassette amplified from the pAW1 plasmid (forward primer: 5′- GTT TCT TAA TTT TAT TTA TTC TTT TCT TGT TCT TTC TTT AGA AAA GAT ATT TTC ATT TAA TTT GTC TCG CGG TTT TTT TTA GTA TAA TAG TCT GTA TAT CCG GAT CCC CGG GTT AAT TAA-3′; reverse primer: 5′- TAT AGG GTC AAT TAT TAA TAT TTA CAA TGT CTA TAA ACA TTA ACT AAA ATT ATT TGA TGT TTG TAC GGA TAT CAA ATA AAA AGT CGA AAA TTC ATT AAT AGA ATT CGA GCT CGT TTA AAC-3′). Deletion cassettes were transformed into JK484 using the lithium acetate method to obtain integrants. Ura + transformants were then isolated and subjected to colony PCR to identify clones in which the construct had integrated into the genome via homologous integration.

The *hos2* “exchange” cassette was constructed by PCR amplifying the *hos2* gene from the pREP1-hos2 plasmid (forward primer: 5′-GGC GGC TCG AGA TGG ATA CTC CTG AGA CAT CCA CAC-3′; reverse primer; 5′- GGC GGG AGC TCT CAG CCT CGA ACG CGA AC-3′). The amplicon was then cloned into the *Xho1* and *SacI* sites of the pAW8X plasmid. The *HDAC3* exchange cassette was constructed by PCR amplifying the HDAC3 cDNA gene from the pOTB7 plasmid (Open Biosystems) (forward primer: 5′- GGC GG C TCG AGA TGG CCA AGA CCG TGG C-3′; reverse primer: 5′-GGC GGG AGC TC T TAA ATC TCC ACA TCG CTT TCC TT-3′). The amplicon was then cloned into the *XhoI* and *SacI* sites of the pAW8X plasmid. Exchange of the *ura4*^+^ gene with either *hos2* or *HDAC3* was achieved according to the protocol of Watson et al. [32].

## Abbreviations

HDAC: Histone Deacetylase; SIN: Septation Initiation Network; YES: Yeast Extract Supplements; EMM: Edinburgh Minimal Media; LatA: Latrunculin A; DMSO: Dimethyl sulfoxide; DAPI: 4′,6-diamidino-2-phenylindole; PBS: Phosphate Buffered Saline.

## Competing interests

The authors declare that they have no competing interests.

## Authors’ contributions

JK designed the experiments and drafted the manuscript. CG, JH, SR, and JK performed the experiments and analysed the data. All authors read and approved the final manuscript.

## Authors’ information

At the time of their contributions CG and SR were MSc candidates at the UWO Department of Biology. CG is currently a PhD candidate at the University of Alberta. SR is currently a PhD candidate at McGill University. JH is a BSc (Hons) student at the UWO Department of Biology. JK is an Assistant Professor at the UWO Department of Biology.

## Supplementary Material

Additional file 1 **Hos2p shares significant sequence similarity to human HDAC3. (A)** Phylogenetic relationship of Hos2p orthologs from yeast to humans. **(B)** Domain structure of fission yeast Hos2p, and human HDAC3. Red diamond indicates conserved Y321 residue necessary for catalysis. **(C)** ClustalW alignment of fission yeast Hos2p, and human HDAC3. Stars indicate amino acid identity and “.” indicates amino acid similarity. Red arrow indicates conserved Y321 residue necessary for catalysis. Click here for file

Additional file 2 **Clp1p and Cdc7p localization upon LatA treatment in *hos2 ****∆ ***backgrounds. (A)** Wild-type and *hos2∆* strains expressing Clp1-GFP were grown to mid-log phase at 30°C and then treated with 0.5 μM LatA for 3 h before fluorescence imaging. Asterisks indicate cells in which Clp1p is enriched in the cytoplasm indicating active SIN signaling. Bar, 10 μm. **(B)** Wild-type and *hos2∆* strains expressing Cdc7-GFP were grown to mid-log phase at 30°C and then treated with 0.5 μM LatA for 3 h before fluorescence imaging. Arrows indicate cells in which Cdc7p localizes asymmetrically to one of two available SPBs indicating active SIN signaling. Bar, 10 μm. Click here for file

Additional file 3 ***clp1 ****∆ ****hos2 ****∆ ***double mutants display increased sensitivity to LatA relative to both*clp1****∆ ***and *hos2 ****∆ ***single mutants. ** Cells of the indicated genotype were grown to mid-log phase at 30°C and then treated with 0.1 or 0.5 μM LatA for 5 h before being fixed and stained with DAPI (nuclei) and aniline blue (cell wall/septa). Bar, 10 μm. Click here for file

Additional file 4 **the *hos2 *gene deletion reduces the restrictive temperature of the *cdc15-140 *mutation. ** Cells of the indicated genotype were streaked to YES plates and incubated over-night At 25°C, 31°C, or 36°C. Bar, 50 μm. Click here for file

Additional file 5 Time-lapse movie of Rlc1-GFP dynamics in wildtype cells treated with DMSO. Click here for file

Additional file 6 Time-lapse movie of Rlc1-GFP dynamics in hos2? cells treated with DMSO. Click here for file

Additional file 7 Time-lapse movie of Rlc1-GFP dynamics in wildtype cells treated with 0.5 µM LatA. Click here for file

Additional file 8 Time-lapse movie of Rlc1-GFP dynamics in hos2? cells treated with 0.5 µM LatA Click here for file

Additional file 9 **Schematic representation of recombinase-mediated cassette exchange. ** A “base-strain” is first created in which the *hos2 * open reading frame is replaced with a deletion cassette composed of the *ura4*^+^ selectable marker flanked by the Cre recombinase recognitions sites, loxP and loxM3. Next, the “exchange” plasmid (pAW8X-*HDAC3*) containing the Cre recombinase gene under control of the nmt41 promoter is transformed into the base strain. Expression of the recombinase permits exchange of the *ura4*^+^ cassette with the *HDAC3* sequence. Click here for file
