# Supplementary figures and images for: A conserved histone deacetylase with a role in the regulation of cytokinesis in Schizosaccharomyces pombe
Source: Cell Div. 2012 May 4;7:13. doi: 10.1186/1747-1028-7-13 (PMC3485120; doi:10.1186/1747-1028-7-13)

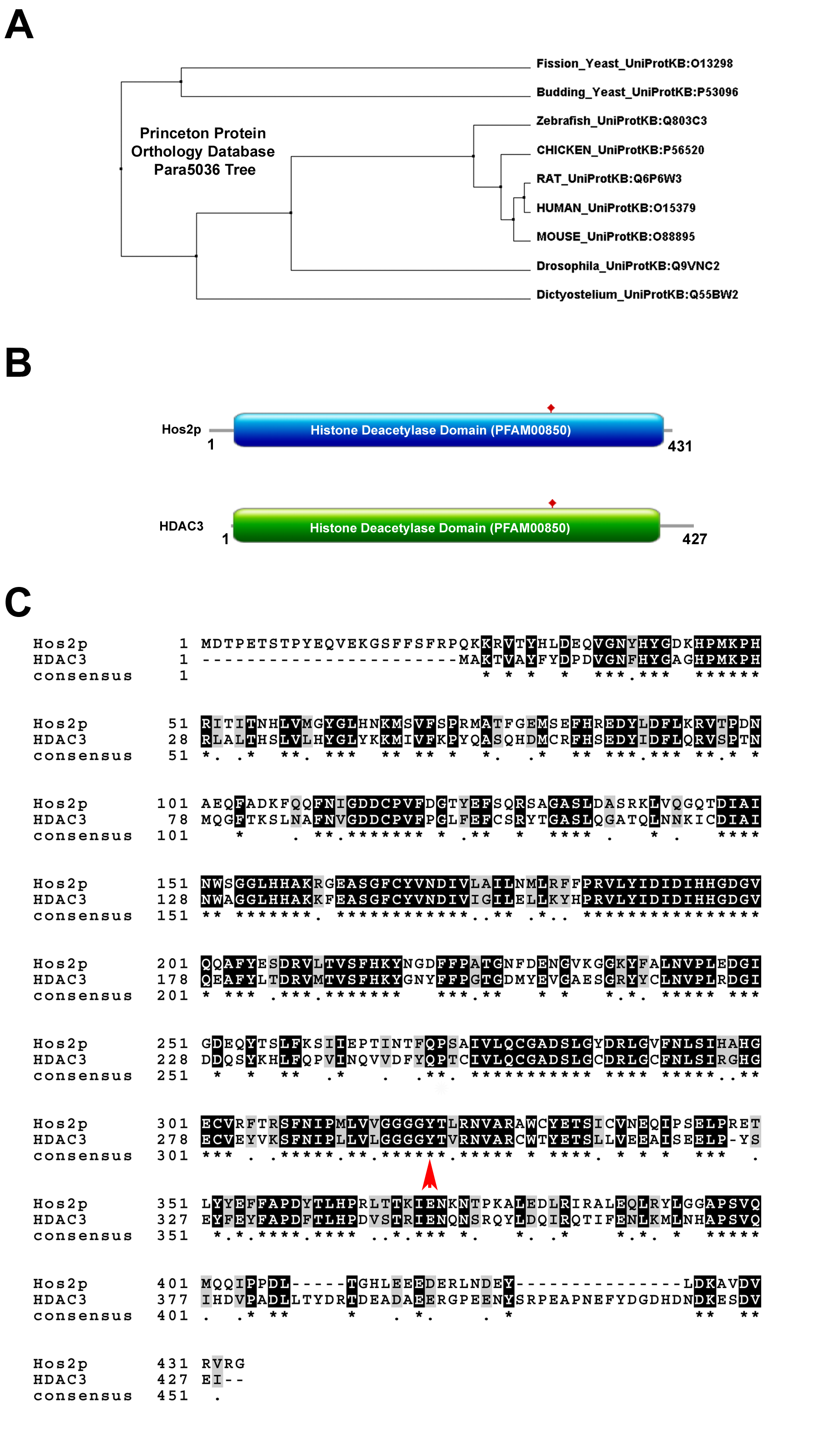

Supplement: Additional file 1 — Hos2p shares significant sequence similarity to human HDAC3. (A) Phylogenetic relationship of Hos2p orthologs from yeast to humans. (B) Domain structure of fission yeast Hos2p, and human HDAC3. Red diamond indicates conserved Y321 residue necessary for catalysis. (C) ClustalW alignment of fission yeast Hos2p, and human HDAC3. Stars indicate amino acid identity and “.” indicates amino acid similarity. Red arrow indicates conserved Y321 residue necessary for catalysis. [file 1747-1028-7-13-S1.tiff]

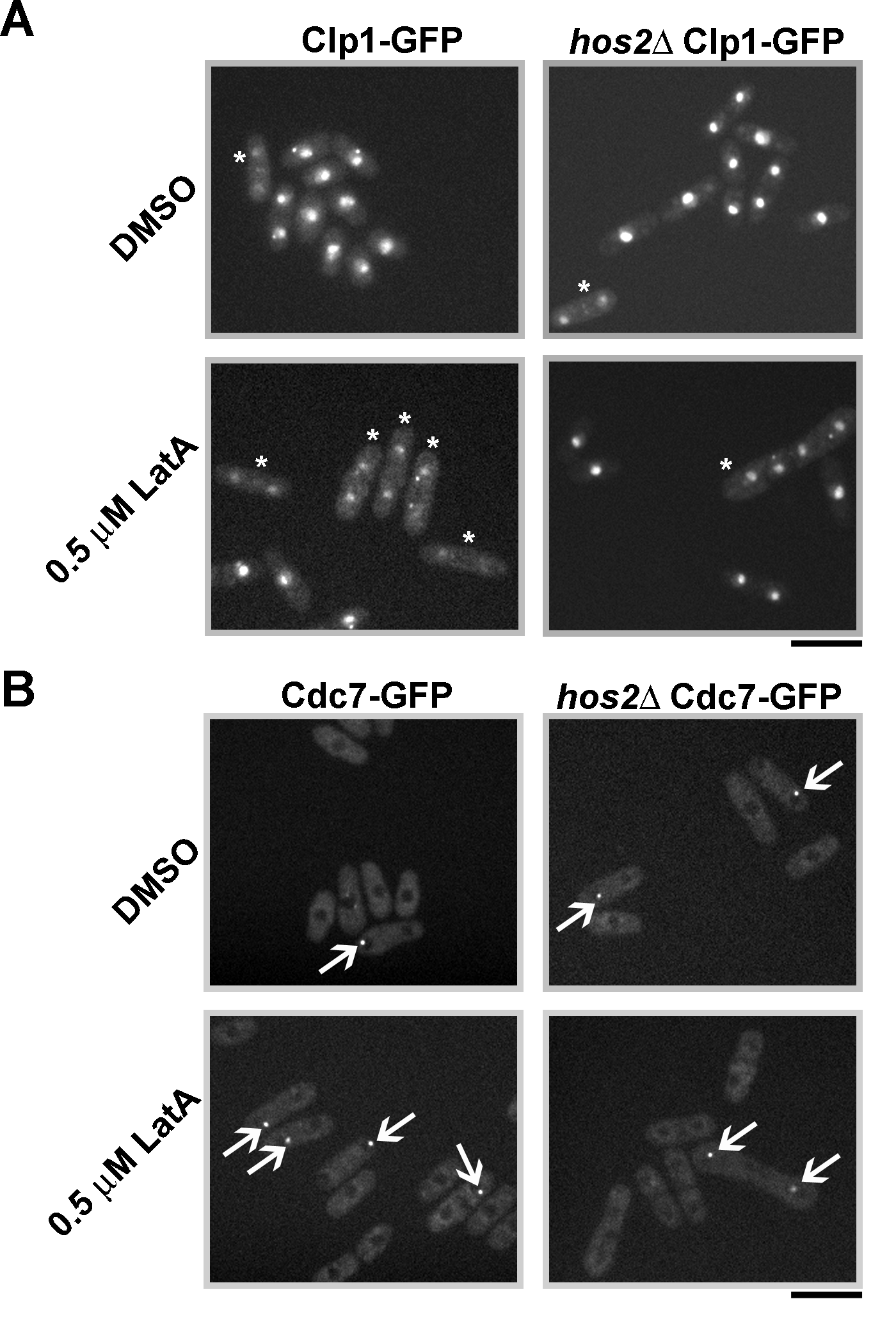

Supplement: Additional file 2 — Clp1p and Cdc7p localization upon LatA treatment in hos2 ∆ backgrounds. (A) Wild-type and hos2∆ strains expressing Clp1-GFP were grown to mid-log phase at 30°C and then treated with 0.5 μM LatA for 3 h before fluorescence imaging. Asterisks indicate cells in which Clp1p is enriched in the cytoplasm indicating active SIN signaling. Bar, 10 μm. (B) Wild-type and hos2∆ strains expressing Cdc7-GFP were grown to mid-log phase at 30°C and then treated with 0.5 μM LatA for 3 h before fluorescence imaging. Arrows indicate cells in which Cdc7p localizes asymmetrically to one of two available SPBs indicating active SIN signaling. Bar, 10 μm. [file 1747-1028-7-13-S2.tiff]

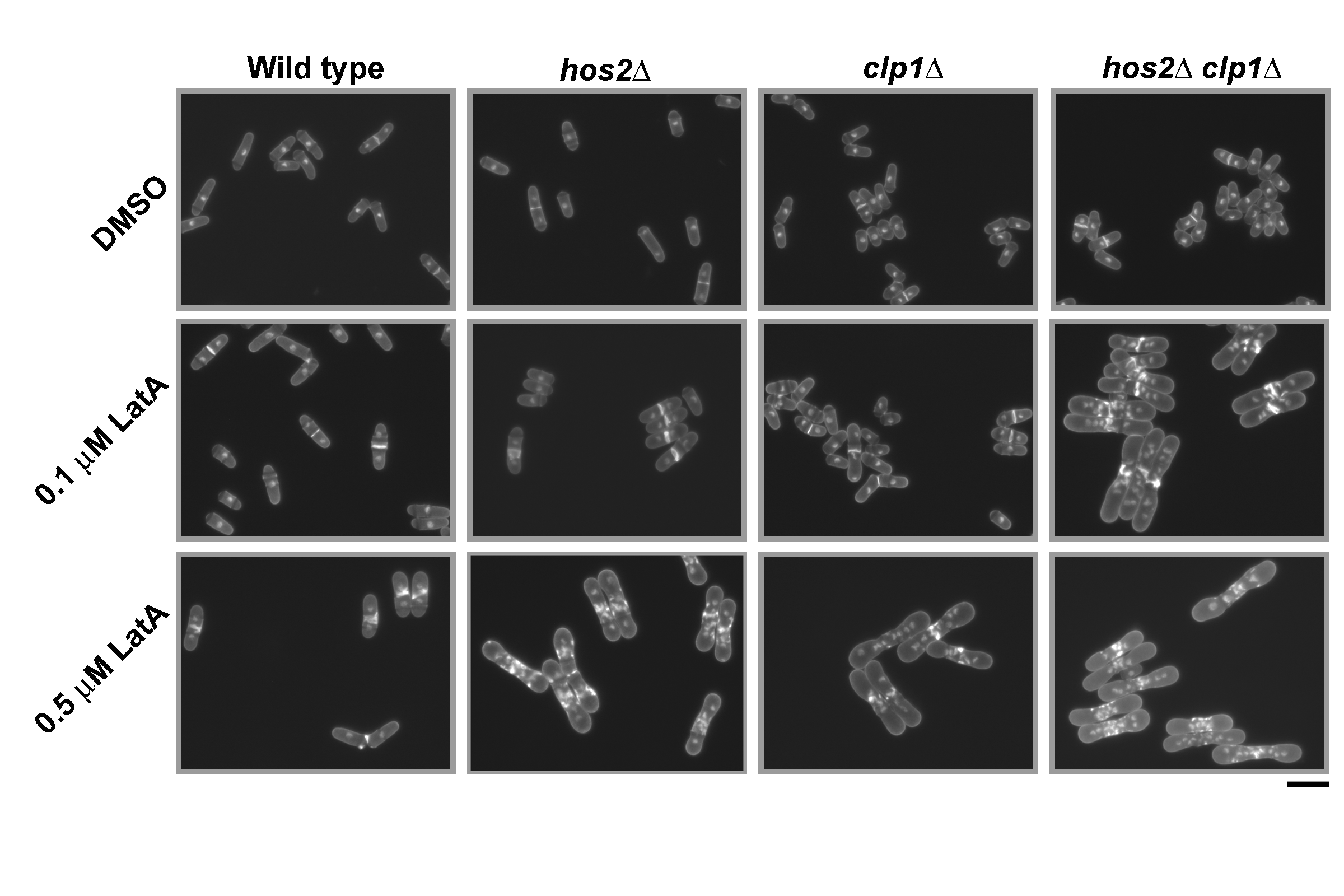

Supplement: Additional file 3 — clp1 ∆ hos2 ∆ double mutants display increased sensitivity to LatA relative to bothclp1∆ and hos2 ∆ single mutants. Cells of the indicated genotype were grown to mid-log phase at 30°C and then treated with 0.1 or 0.5 μM LatA for 5 h before being fixed and stained with DAPI (nuclei) and aniline blue (cell wall/septa). Bar, 10 μm. [file 1747-1028-7-13-S3.tiff]

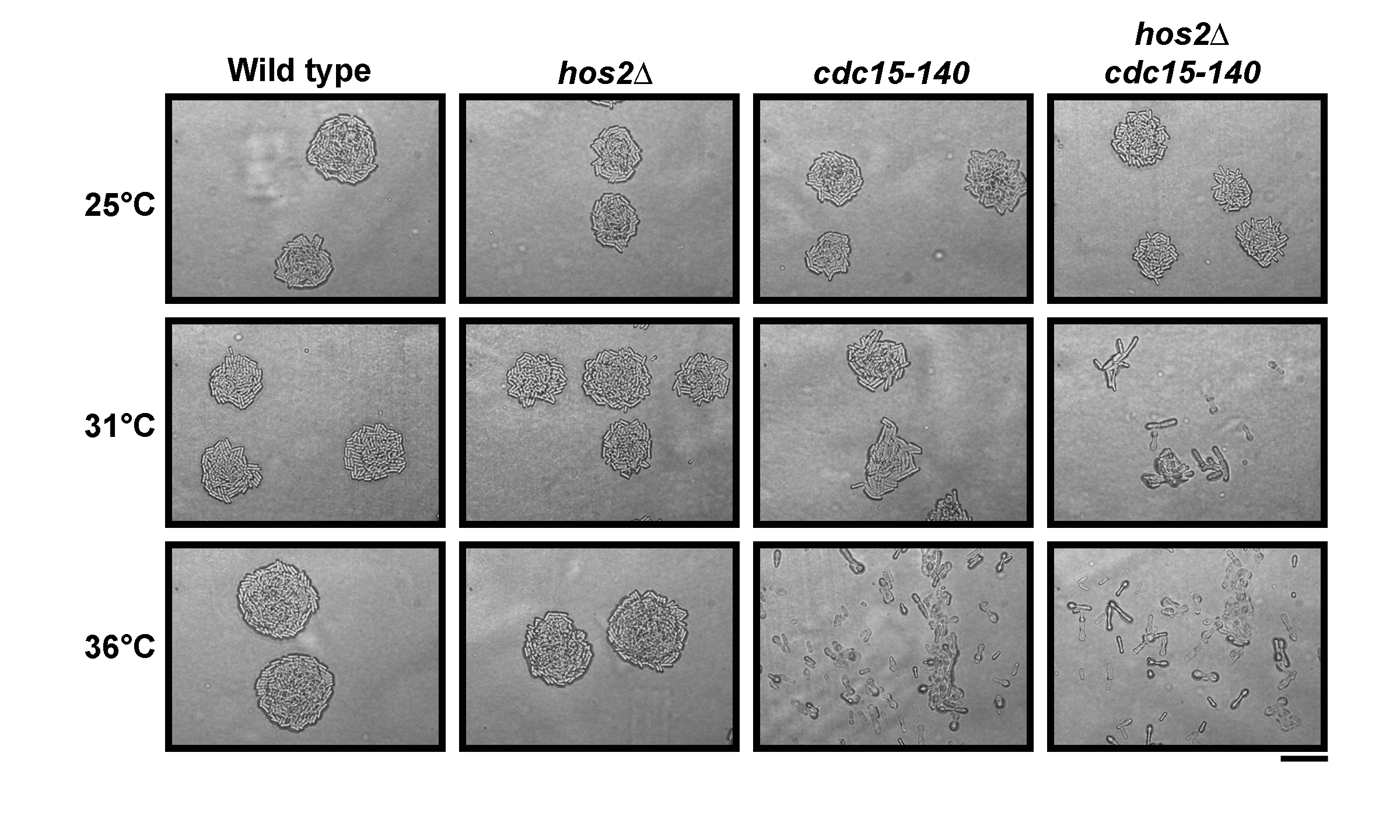

Supplement: Additional file 4 — the hos2 gene deletion reduces the restrictive temperature of the cdc15-140 mutation. Cells of the indicated genotype were streaked to YES plates and incubated over-night At 25°C, 31°C, or 36°C. Bar, 50 μm. [file 1747-1028-7-13-S4.tiff]

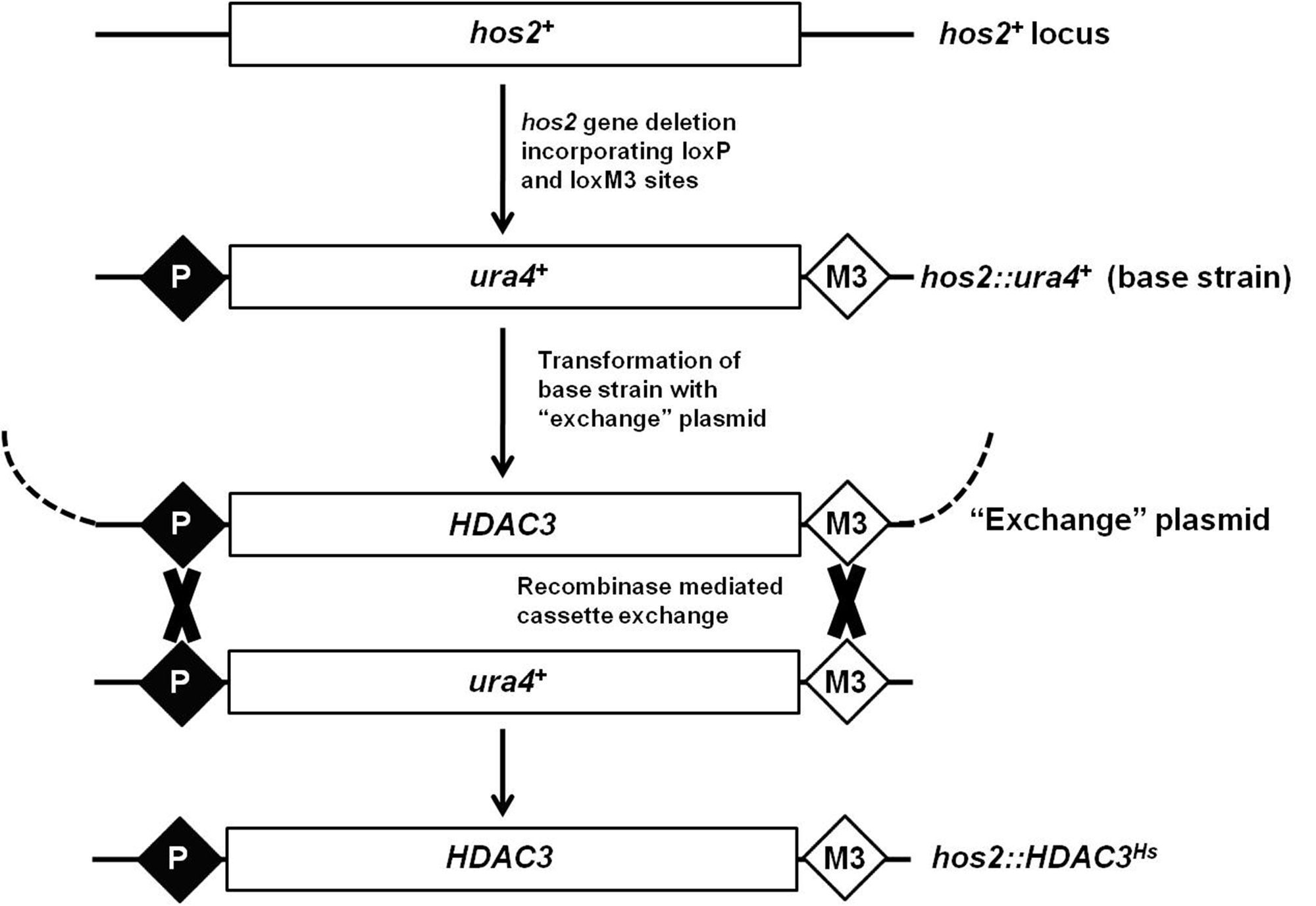

Supplement: Additional file 9 — Schematic representation of recombinase-mediated cassette exchange. A “base-strain” is first created in which the hos2 open reading frame is replaced with a deletion cassette composed of the ura4+ selectable marker flanked by the Cre recombinase recognitions sites, loxP and loxM3. Next, the “exchange” plasmid (pAW8X-HDAC3) containing the Cre recombinase gene under control of the nmt41 promoter is transformed into the base strain. Expression of the recombinase permits exchange of the ura4+ cassette with the HDAC3 sequence. [file 1747-1028-7-13-S9.tiff]
